# Using Patient and Family Engagement Strategies to Improve Outcomes of Health Information Technology Initiatives: Scoping Review

**DOI:** 10.2196/14683

**Published:** 2019-10-08

**Authors:** Kevin Leung, Drew Lu-McLean, Craig Kuziemsky, Richard G Booth, Sarah Collins Rossetti, Elizabeth Borycki, Gillian Strudwick

**Affiliations:** 1 Centre for Addiction and Mental Health Toronto, ON Canada; 2 McMaster University Hamilton, ON Canada; 3 University of Ottawa Ottawa, ON Canada; 4 Western University London, ON Canada; 5 Columbia University New York, NY United States; 6 University of Victoria Victoria, BC Canada

**Keywords:** health information technology, informatics, patient engagement, medical informatics, health services

## Abstract

**Background:**

Many health care organizations around the world have implemented health information technologies (ITs) to enhance health service efficiency, effectiveness, and safety. Studies have demonstrated that promising outcomes of health IT initiatives can be obtained when patients and family members participate and engage in the adoption, use, and evaluation of these technologies. Despite knowing this, there is a lack of health care organizations using patient and family engagement strategies to enhance the use and adoption of health ITs, specifically.

**Objective:**

This study aimed to answer the following three research questions (RQs): (1) what current frameworks or theories have been used to guide patient and family engagement in health IT adoption, use, implementation, selection, and evaluation?, (2) what studies have been done on patient and family engagement strategies in health IT adoption, use, implementation, selection, and evaluation?, and (3) what patient and family engagement frameworks, studies, or resources identified in the literature can be applied to health IT adoption, use, implementation, selection, and evaluation?

**Methods:**

This scoping review used a five-step framework developed by Arksey and O’Malley and adapted by Levac et al. These steps include the following: (1) identifying the RQ, (2) identifying relevant studies, (3) selecting studies, (4) charting relevant data, and (5) summarizing and reporting the result. Retrieved academic and grey literature records were evaluated using a literature review software based on inclusion and exclusion criteria by two independent reviewers. If consensus was not achieved, two reviewers would resolve conflicts by discussion. Research findings and strategies were extracted from the studies and summarized in data tables.

**Results:**

A total of 35 academic articles and 23 gray literature documents met the inclusion criteria. In total, 20 of the 35 included studies have been published since 2017. Frameworks found include the patient engagement framework developed by Healthcare Information and Management Systems Society and the patient and family engagement framework proposed by Carman et al. Effective strategies include providing patients with clear expectations and responsibilities and providing reimbursement for time and travel. The gray literature sources outlined key considerations for planning and supporting engagement initiatives such as providing patients with professional development opportunities, and embedding patients in existing governance structures.

**Conclusions:**

Several studies have reported their findings regarding successful strategies to engage patients and family members in health IT initiatives and the positive impact that can emerge when patients and family members are engaged in such initiatives in an effective manner. Currently, no framework has consolidated all of the key strategies and considerations that were found in this review to guide health care organizations when engaging patients and family members in a health IT–specific project or initiative. Further research to evaluate and validate the existing strategies would be of value.

## Introduction

Globally, health care organizations have implemented health information technologies (ITs) to improve health service efficiency, effectiveness, and patient safety [1-9]. Health IT in this context refers to several technologies implemented in clinical settings to improve an aspect of clinical care. These technologies may include but are not limited to (1) electronic health records (EHRs; medical records in electronic form), (2) patient portals (Web access to a variety of functions such as viewing lab results and booking an appointment), (3) care coordination portals (electronic portals that support the coordination of care activities), and (4) mobile health apps (apps to track, learn, or monitor an aspect of one’s health) [10,11]. Some organizations have implemented patient portals that are tethered to hospital EHRs to provide patient access to their health information and provide patients with digital tools to take more control over their own health and care [12-14]. However, not all health care organizations have consistently taken full advantage of their health IT and systems [15]. Therefore, many of their potential benefits to patients, families, health professionals, and the health care system remain unseen [15].

Several studies have shown promising results from health IT when engaging end users such as patients and family members in the adoption, use, and evaluation of a specific health IT such as an EHR [16,17]. One article revealed a decrease in medication errors at the hospital when patients were engaged in the implementation of an electronic medication administration system [16]. By engaging patients in interviews to determine the best methods for identifying mental health patients through the barcode scanning process, an improvement in medication scanning rates and reduction in medication errors was realized [7]. In another study, patients engaged in user interface testing of a patient portal improved the usability and uptake of the technology by marginalized patient populations [18]. These examples show that active ways (eg, incorporating patients on design teams and obtaining patient feedback preimplementation of a health IT) of engaging patients and family members in health IT activities can be beneficial [18]. If health care organizations begin to effectively engage their patients and families in the development, adoption, use, and evaluation of health IT more regularly, there may be an increasing number of benefits realized.

Engaging patients and family members in decision making related to health services is not a new concept [19]. In fact, patients have engaged with health care organizations in many ways for several years now, such as advising health care institutions about health service delivery and patient-oriented health research [20]. In the United States, the Patient-Centered Outcomes Research Institute has advocated and funded the engagement of patients in many research initiatives [21]. However, patient engagement in health IT development, adoption, use, and evaluation is not as mature as it is in patient participatory medicine for health service delivery. The term, *patient engagement*, in this context broadly encompasses processes that involve informing and/or partnering with patients [22], to inform health service planning and delivery of the health IT. Engaging in a patient’s own health care is outside of the scope of this discussion.

The objective of this scoping review was to explore studies that aim to improve outcomes of health IT initiatives through patient and family engagement and to explore practical strategies that health care organizations can leverage to meaningfully engage patients and families across the continuum of health IT initiatives. The review specifically focuses on how patients and family members can be involved in the health service planning and delivery context and not how they are more engaged in their own care.

## Methods

This scoping review adopted the 5-step framework defined by Arksey and O’Malley [23] and updated by Levac et al [24]. These steps include the following: (1) identifying the research question (RQ), (2) identifying relevant studies, (3) selecting studies for data collection, (4) charting relevant data, and (5) summarizing and reporting the result [23,24]. Ethical approval was not required for this study. The following sections outline the steps followed to conduct the review.

### Step 1: Identifying the Research Questions

The literature review was conducted to answer the following three RQs:

- RQ1: What current frameworks, models, or theories have been used to guide patient and family engagement in health IT adoption, use, implementation, selection, and evaluation?
- RQ2: What studies have been done on patient and family engagement strategies in health IT adoption, use, implementation, selection, and evaluation? And what are their results?
- RQ3: What patient and family engagement frameworks (not specific to health IT), studies, and/or resources can be applied to health IT adoption, use, implementation, selection, and evaluation?

The use of the 3 RQs was done to clearly articulate the scope of the review and explore literature that can be applied broadly to patient and family engagement in health IT projects or initiatives. The following assumptions were made to clarify the terminology used in the study when developing the RQs. The broader term *health IT* was used to represent various forms of IT used in health contexts such as (but not limited to) *EHRs* (patient records accessible via a computer) [25], *patient portals* (secure websites that allow patients to access their health record and other functions such as booking a medical appointment) [26], *care coordination portals*, and *mobile health apps* (use of mobile devices to provide health care services) [27]. RQs and terminology were refined through consultation with the members of the research team and a patient and family advisory committee. The patient and family advisory committee was based at a Canadian hospital located in Toronto, Ontario.

### Step 2: Identifying Relevant Studies

With the guidance of a research librarian with experience conducting scoping reviews, a search strategy was developed using the following databases: MEDLINE, PsycINFO, Culmulative Index for Nursing and Allied Health Literature, Theses Canada, and the Education Resources Information Center. These databases are all commonly used in health science–related literature reviews with a focus on health services and were made available through the organizations in which the authors are employed. A primary search strategy was developed for the MEDLINE database (see Table 1) and adapted to be used for the other electronic databases.

The search strategy used a combination of relevant Medical Subject Headings terms identified by the research librarian and keywords with Boolean terms (search combinations are shown in Table 1 via the use of the various search syntax shown above). The database search was supplemented with a search for unpublished gray literature related to patient engagement frameworks and toolkits. The search tool by the Canadian Agency for Drugs and Technologies in Health, Grey Matters, guided the gray literature search. A search using Google’s search engine was also completed. Additional methods for identifying relevant resources included the following: (1) reference searching from key article reference lists, (2) input from health IT experts, and (3) the patient and family advisor committee.

The gray literature search used keywords to refine the search that included the following: ehealth, electronic medical records, electronic health records, patient portals, patient/families/caregiver engagement, ehealth adoption, use and evaluation, frameworks/ strategies/ resources/ tools/ toolkits, and health IT. Searches were completed between June and December 2018, and the screening process took part in early 2019.

#### Inclusion/Exclusion Criteria

Studies and frameworks in any clinical or health care setting were included. The studies and documents assessed were not limited to their date of publication or country of origin. Studies not published in English were excluded from this review. Systematic reviews were not eligible, but their reference lists were screened to find supplementary relevant studies. Studies that did not explicitly address patient or family engagement were excluded from the review. The focus of the review was on studies that involved, engaged, or empowered patients, families in the decision-making process, and across all stages of a health IT initiative for health service planning and delivery and not studies that focused on technologies that can be used to engage patients, families, and/or caregivers in their care. Studies that focused on patient engagement as an outcome of integrating and enabling technologies in the care process were, therefore, excluded.

**Table 1:** Shows the search strategy developed for the MEDLINE database.

No	Searches	Results
1	health records, personal/or patient portals/	1531
2	exp Medical Records Systems, Computerized/ or exp Electronic Health Records/ or exp Hospital Information Systems/ or exp Information Systems/	235,853
3	exp Patient Participation/	23,426
4	((patient or family or caregiver) adj2 (engag* or involv* or empower* or activat* or participat*)).mp.	46,576
5	framework.ab,ti.	217,304
6	exp Telemedicine/	24,262
7	exp Patient Portals/	179
8	“patient and public involvement”.kw.	145
9	exp Medical Informatics/	419,180
10	theory.ab,ti.	303,436
11	model.ab,ti.	1,870,340
12	Search #5 or Search #10 or Search #11 [framework set]	2,259,697
13	3 or 4 or 8 [patient engagement set]	46,658
14	Biomedical Technology/	5923
15	Search #1 or Search #2 or Search #6 or Search #7 or Search #9 or Search #14 [eHealth set]	472,799
16	Search #12 and Search #13 and Search #15	558

### Step 3: Study Selection

Numerous records were retrieved from the academic database search and gray literature search. Literature screening was completed using the Covidence systematic review software [28]. The software removed duplicates from the database search to aid with screening. Once duplicates were removed, 2 members of the research team independently screened the article titles and abstracts to determine if the full-text article should be retrieved and assessed. After the title and abstract screening, the inter-rater reliability score was calculated. Conflicts/discrepancies between screeners were resolved by discussion between the 2 screeners, and if consensus was not reached, a third member of the research team was consulted.

### Step 4: Charting the Data

From each included study or gray literature document, pertinent information was extracted and summarized to address the RQs. This information included the following: descriptions of the study (study name, authorship, country of publication, journal published, study design, and population study), study methods (engagement strategies employed by researchers), and all study results related to the RQs (lessons learned and recommended engagement strategies).

### Step 5: Collating, Summarizing, and Reporting Results

Results were collated, summarized, and reported based on the RQ or RQs that the article addressed. Descriptive statistics were used to summarize characteristics of the articles found through the database and gray literature search. A content analysis was performed on the identified studies to identify and record overarching themes that emerged. The approach to categorizing themes was an iterative, inductive process involving 2 members of the research team. The 2 members of the research team met after the data extraction process to create and refine the overarching themes identified. The recommended patient engagement strategies and considerations discussed in the included articles were also recorded.

## Results

### Characteristics of the Identified Studies

**Figure 1:**
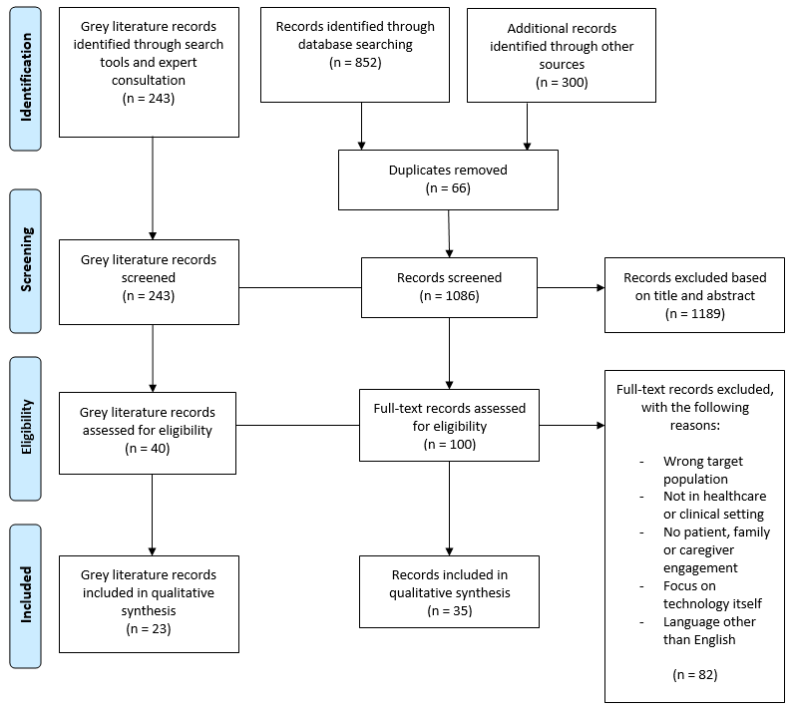
Preferred Reporting Items for Systematic Reviews and Meta-Analyses diagram indicating the flow of records reviewed during the literature search.

A total of 1395 academic literature records, or gray literature documents, were retrieved from the academic and gray searches and expert consultation. During the abstract and title screening process, the inter-rater reliability between the 2 independent raters (KL/DM) was above 75% (Cohen kappa=.44). After the full-text records were assessed, 35 (n=35) academic articles and 23 (n=23) gray literature documents met the inclusion criteria (see Figure 1). The publication date of the academic articles and gray literature ranged between 2005 and 2018. The studies were conducted in the United States (n=14), and the remaining publications originated in other countries located in North America, Europe, and Asia. From the gray literature, 13 (n=13) documents were published in Canada, 4 (n=4) in Australia, 3 (n=3) in the United Kingdom, and 3 (n=3) in the United States. Table 2 provides an overview of the design of the academic and gray literature included in this review. Multimedia Appendix 1 provides the full data extraction table for all included records.

**Table 2:** Design of the identified academic and gray literature (N=58).

Design	Statistics, n (%)
Quantitative	1 (2)
Qualitative	21 (36)
Mixed methods	1 (2)
Other academic papers (eg, case reports and editorials)	12 (20)
Reports/websites/other forms of gray literature	23 (40)

### RQ1: Current Health Information Technology–Related Frameworks, Models, or Theories

A total of 2 articles outlined frameworks or models that have been used to effectively guide patient and family engagement in health IT adoption, use, implementation, selection, and evaluation [29,30]. In the first article that addresses RQ1, the Healthcare Information and Management Systems Society patient engagement framework was discussed. This framework was created to inform health care organizations on how to leverage health IT systems to implement patient engagement strategies [29]. The framework outlines 5 approaches to engagement that align with the International Association for Public Participation’s spectrum for community engagement and suggest tools such as patient-accessible records and patient-specific education to inform and partner with patients. Although these tools are not at the health service delivery level, the stages of engagement discussed in the framework are applicable to health IT health service planning and delivery. These stages of engagement are as follows: (1) inform, (2) consult, (3) involve, (4) collaborate, and (5) empower [22].

The second article that addressed RQ1 is a study by Walker et al that outlined an evaluative model for health IT implementation [30]. Walker et al engaged patient groups through a series of focus groups and an online forum to understand the evaluation needs of patients related to an inpatient portal implementation. Through a nominal group approach, patients specified the following 2 categories of patient-specific outcomes that should be incorporated in an evaluation of an inpatient portal: (1) outcomes related to the explicit use of the inpatient portal and (2) outcomes related to the tacit knowledge gained by having access to an inpatient portal [30]. Explicit use of the inpatient portal may result in a change in patient satisfaction, which can be a measure of patient outcomes as a result of portal implementation and use [30]. Tacit knowledge that is gained from using the inpatient portal could result in change in patient engagement in health IT, which can be a measurable outcome [30]. The model demonstrates that soliciting patient feedback on explicit portal use and changes in tacit knowledge is needed for a multifaceted evaluation of an inpatient portal implementation.

### RQ2: Patient and Family Engagement Strategies in Health Information Technology Initiatives

A total of 19 (n=19) studies were identified that utilized patient and family engagement strategies in health IT adoption, use, implementation, selection, and evaluation. A finding of 2 studies related to the use of engaging patients through multiple digital modalities. A study by Athilingam et al engaged patients on the design, potential features, and the ease of use of a mobile health app to improve self-care in heart failure [31]. Interviews were conducted with 10 patients and 4 cardiologists before the initial prototype was developed. The authors concluded that existing mobile health apps have not been widely adopted and suggested the mixed use of internet, email, and text messages for promoting better communication and long-term engagement with digital health apps [31]. Another study by Lafata et al reiterated the need for different digital modalities to effectively engage patients from vulnerable patient populations [32].

#### Multidisciplinary, Team-Based Approach

A total of 4 (n=4) studies suggested that a multidisciplinary, team-based approach would be an effective engagement strategy for engaging patients and families in the use of health IT [33-36]. A study by Ackerman et al examined the patient engagement strategies during the implementation of a patient portal at 5 community health centers [33]. Portal champions reminded clinicians and staff to encourage patients to sign up for the portal. Volunteers, front desk clerks, and medical assistants provided enrollment assistance to patients and used clinic computers to demonstrate to patients how to use portal services. The study by Ackerman et al found that the uptake and use of the portal improved when patients were engaged by trusted staff members or clinicians. Results from the study by Raval et al concluded that the engagement of a pediatric surgeon and physician assistant was crucial to the success of recruiting and engaging patients in the development of a mobile app for colorectal disease management [34]. As the clinical team was available during the planning stages of the research project, it helped ease the process of engaging patients in the study. As a result, pediatric patients and their family members who were already visiting the hospital were successfully recruited and provided useful feedback. Similar recommendations were made by Krist et al and Shapiro-Mathews et al [35,36]. In a study by Krist et al, patient engagement strategies in primary care practices were evaluated [35]. Primary care offices had registration staff pass out information to patients, had nurses discuss how to sign up for a patient portal, and other clinicians communicate the value of signing up for the patient portal. Krist et al concluded that a team-based approach to engaging patients positively influenced the uptake of the patient portal compared with the clinician-dependent approach to engaging patients. The article by Shapiro-Mathews et al outlined an institutional strategy for mobile health technologies that requires an interdisciplinary approach [36]. A clinical nurse specialist and other health care professionals can facilitate a team-based approach to engage patients, provide patient education, and inform the design of mobile apps that meet the needs of patients.

#### Training/Education of Patients

A total of 8 (n=8) articles highlighted the importance of training/education of patients in the success of health IT adoption, use, implementation, selection, and evaluation. The results from a study by Anshari et al showed that the availability of a Web-based health educator is important to improving the health literacy of patients and empowering patients to control their own health and health information [37]. Greysen et al conducted a randomized controlled trial where patients in the intervention arm received tailored, structured education regarding the use of a patient portal at the bedside [38]. Study results suggested that bedside portal training produced a trend of increased ability to log in and navigate the portal, satisfaction with portal use, and frequency of portal use after discharge. Van den Bulck et al explored patient needs, expectations, and attitudes toward a patient portal by administering an online survey to recruited patients [39]. Results showed that digital health literacy is a key factor to portal adoption and providing training to patients could provide exposure to using the portal and create appropriate expectations of what the portal is capable of. Another study by Wildenbos et al explored experiences and perspectives of older adult patients using a patient portal 1-year postimplementation of the technology [40]. The results from the study by Wildenbos et al concluded that health literacy level of patients was a strong factor that influenced the patient’s overall interest and perceived ability to use the patient portal. Although the previously discussed training/education methods may be most directed at engaging patients in their own care, similar methods could be used to orient patients before engaging in a health IT initiative aimed at supporting adoption, use, implementation, selection, and evaluation.

#### Training of Health Care Providers

In addition to training patients on the use of health IT, the study by Raval et al also recommended that health care providers may need to be trained to address potentially low levels of self-efficacy of employing effective patient engagement methods [34]. Wildenbos et al also emphasized the need for health care providers to be trained regarding how to effectively engage patients [41]. Likewise, a study by Metting et al explored patient needs and opinions through focus groups to facilitate the development of patient Web portals [42]. A training activity that was recommended included training health care providers to effectively communicate with patients with regards to engagement specific activities.

### RQ3: General Patient and Family Engagement Frameworks, Studies, and Resources

A total of 13 (n=13) academic sources and 23 (n=23) gray literature sources were found that employed patient and family engagement strategies for research and clinically relevant projects that were not health IT–specific, but the principles or findings embedded within them could potentially be applied to this context. In turn, relevant principles and findings may be included in a future health IT–specific patient and family engagement framework or set of recommendations for health care organizations to utilize. Of the 13 academic articles, 6 (n=6) explicitly outlined frameworks that can be used to involve patients and family members within nonhealth IT contexts such as research, health service delivery, and quality improvement.

**Figure 2:**
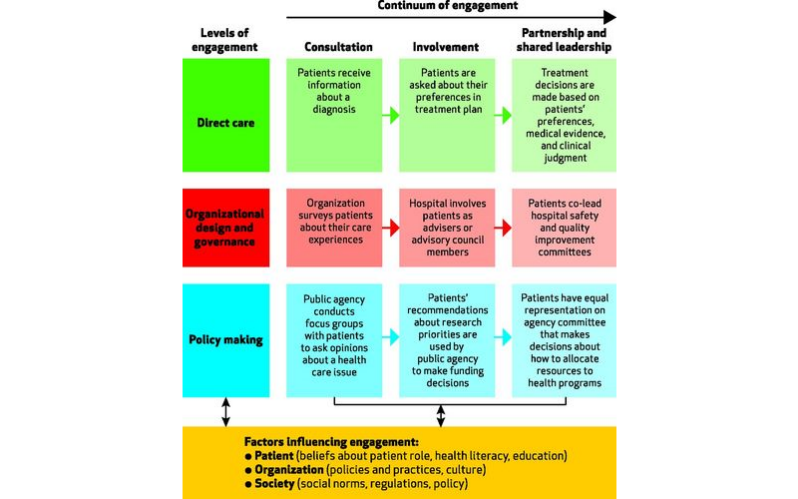
Multidimensional framework for patient and family engagement in health and health care (permission from author and publisher obtained to use image, and copyright obtained).

A framework proposed by Carman et al was widely used to inform effective patient and family engagement strategies in health IT adoption, implementation, use, selection, and evaluation [43] (see Figure 2). The framework outlines 3 categories of engagement activities across a continuum: (1) consultation, (2) partnership, and (3) shared leadership. These 3 categories can be applied in different levels of the health care system, which was segmented by the authors as follows: individual care, organization governance, and government policy. The framework highlighted individual factors that can potentially impact a patient’s willingness and ability to engage with the health care system such as health literacy and education level. At the institutional level, health care organizations and staff can also encourage patient engagement through demonstrating that patient participation and leadership is imperative to the achievement of organizational goals [43].

Policies and practices that can influence patient engagement can create expectations that patients can and will serve as advisors and decision makers on committees and patient-centered councils. Bridgepoint Hospital in Toronto, Canada, implemented practices that brought patients, families, and hospital staff together to redesign health care services at the hospital and improve the overall patient experience [44]. Patients and family members at the hospital were recruited as advisors to review the hospital quality improvement processes.

At the government level, policy makers can create mechanisms so that patients can provide input in public policy, such as public deliberation sessions, town hall meetings, public hearings, or regulatory comment processes [43]. Nonprofit organizations and government agencies can also aid in creating funding mechanisms requiring patient participation in the decision-making process. These efforts to encourage patients and family member participation in quality improvement processes broadly can be applied specifically to the health IT context, creating mechanisms that encourage patients and families to provide their input in health IT–related projects and policies.

A number of the identified studies that addressed RQ3 utilized different strategies to engage patients in a clinical research network or to engage the youth in projects led by academic researchers (see Table 3).

Guidance documents and reports identified in the gray literature highlighted key attributes to patient engagement broadly in the health care and research context. Gray literature sources highlighted key considerations to guide health care organizations in preparing for engagement activities (see Table 4).

**Table 3:** A list of common strategies used in identified academic studies to engage patients and family members.

Recommended strategies	Identified studies
Provide stakeholders with clear expectations, roles, and responsibilities (eg, number of hours and anticipated deliverables)	Perfetto et al (2018) [19], Arkind et al (2015) [45], Boyer et al (2018) [46], Hawke et al (2018) [47], Hamilton et al (2018) [48], Shelef et al (2018) [49], Warren et al (2018) [50]
Develop policy or practice that provides incentive or compensation to stakeholders for their time and efforts (food, travel, reimbursement for time, and provision of training opportunities)	Perfetto et al (2018) [19], Arkind et al (2015) [45], Hawke et al (2018) [47], Hamilton et al (2018) [48], Shelef et al (2018) [49], Warren et al (2018) [50]
Engage stakeholders early in the planning and development stage of the project	Perfetto et al (2018) [19], Arkind et al (2015) [45], Boyer et al (2018) [46], Chung et al (2018) [51], Faulkneret al (2018) [52]
Be transparent about patient contributions being used and making an impact on the project	Perfetto et al (2018) [19], Hawke et al (2018) [47], Chung et al (2018) [51], Coathup et al (2016) [53]
Prioritize effective communication with regular updates and provide explanation of research/medical terminology and show that patients are being valued as partners	Boyer et al (2018) [46], Chung et al (2018) [51], Faulkneret al (2018) [52], Coathup et al (2016) [53]
Allow stakeholders to meet in a convenient time (eg, weekday evenings) and location (eg, remote access)	Perfetto et al (2018) [19], Arkind et al (2015) [45], Hawke et al (2018) [47], Hamilton et al (2018) [48]
Engage a group of more than 2 patients so that they can support one another and have shared discussion	Perfetto et al (2018) [19], Hamilton et al (2018) [48], Faulkneret al (2018) [52]
Leverage clinical providers as trusted agents	Boyer et al (2018) [46], Chung et al (2018) [51], Suarez-Balcazar (2005) [54]
Provide adequate preparation (orientation, training, and resources) for both the engaged stakeholders and the team members engaging stakeholders	Boyer et al (2018) [46], Suarez-Balcazar (2005) [54]
Use established networks of stakeholder groups	Perfetto et al (2018) [19], Boyer et al (2018) [46]
Identify concepts that may be confusing and set aside time to explain them in jargon-free terms	Hawke et al (2018) [47], Hamilton et al (2018) [48]

**Table 4:** Key considerations from existing engagement frameworks and toolkits when preparing for engagement.

Considerations when preparing for engagement	Identified studies
Clarify objectives and impact of engagement activities	[55-57]
Clarify why stakeholders can get involved	[21,58-61]
Discuss preferences of ongoing communication to ensure that all stakeholders involved are informed throughout	[62,63]
Determine the appropriate level of engagement that meets patient and organizational goals	[64]
Clarify and document roles, responsibilities, and scope of engaged population that are agreed upon between project team and engaged population	[58,59,63,65]
Define time commitment and expectations from the engagement team	[55,56,58,60,62]
Consider potential barriers for engagement	[62]
Plan to protect patient privacy	[56]
Involve patients in the planning process	[21,56,57,59,64,66]
Allocate financial resources to reimburse participants for expenses incurred while being involved in project (eg, time, travel, training, translation, and childcare)	[56,58-60,63,67,68]

Additional considerations and characteristics were highlighted to support patients, family members, and staff in health care organizations during engagement activities (see Table 5). In addition to preparing and supporting engagement, 9 (n=9) gray literature sources highlighted the importance of conducting an evaluation to ensure that initiatives to engage patients and family members are meaningful and provide value for all stakeholders [21,57,59,61-65,67]. Studies encouraged the use of quantitative measures and measuring against specified objectives to demonstrate the value of engagement [57,67]. Different evaluative methods were described such as providing evaluation forms, conducting surveys, and engaging in discussions with patients to provide the opportunity for giving constructive feedback. The use of surveys and feedback forms allows for organizations to solicit feedback anonymously, which may be preferred by some patients and family members [62,63,69].

**Table 5:** Key considerations to support patients and family members during engagement activities outlined by gray literature sources.

Considerations when supporting engagement activities	Identified studies
Meet patients/families in their own environments and communities	[59,60]
Schedule meetings at a variety of different times (not just during working hours)	[59]
Allow for family presence when engaging patients	[66]
Engage 2 or more patients at a time	[56]
Provide training and support for stakeholders to effectively communicate and partner with each other	[58,60,62]
Leverage Web-based tools when providing training for patient engagement	[68]
Ensure researchers/project leads have the necessary skills to involve patients	[58,59]
Provide patients with background information/readings before preparing for meetings	[56,63]
Provide patients with opportunities for professional development (eg, authoring manuscripts and presenting at conferences)	[21,60,62,67]
Embed patient partners in existing governance structures (eg, boards, steering committees, advisory councils, and patient groups)	[55,64,67,70]
Communicate in jargon-free language	[57]
Track and update clear timelines of each milestone	[68]
Leverage health care professionals and their ability to encourage patients to engage	[71]
Clearly articulate the type of patient information being shared and why	[56]
Be transparent about the constraints and why input may not be always used	[58,59,61,63,68,72]
Outline outcomes that are important to patients and can improve their quality of life	[55]
Frequently check in with patients for any questions and to keep them informed (eg, through progress reports or newsletters)	[62]
Provide opportunity for stakeholders to use multiple channels of communication (eg, written communication, emails, phone calls, and social media)	[59,63,70]
Provide thank you letters, along with feedback and suggestions for future involvement	[68]

## Discussion

### Primary Findings

Patient and family engagement has become a topic of increasing interest in the research community in the last several years. In fact, the majority (57%, 33/58) of the included studies in this review have been published since 2017. Identified studies have highlighted the importance of involving and partnering with patients in health IT design and development [51,73]. Several studies have used a variety of strategies and outlined considerations when engaging patients and family members in health IT–related projects. These studies outlined several considerations for engaging patients and families, mainly when preparing for engagement or in the early stages of the engagement process (see Table 4). Strategies that were consistent among the identified studies include providing incentives and reimbursement for patients, clarification of patient roles and responsibilities, and demonstrating the value of engagement to patients. However, there is a limited amount of literature that have established a framework, model, or theory, or set of recommendations that can be used to guide patient and family engagement in health IT initiatives. As a result, there is no standardized methodology or resource for engaging patients and family members effectively for health IT initiatives. The development of a practical framework (borrowing concepts, recommendations, and relevant principles from the nonhealth IT literature) to guide health care organizations and health care providers within this unique context is needed. One main limitation for the development of a standardized framework is that it may not capture the unique needs of all diverse patient groups. Therefore, additional resources may be required to supplement and guide effective patient and family engagement for health IT initiatives; however, starting with a resource that health care organizations can use to engage in this important work, is needed.

Patient engagement is a broad term that can be defined by varying levels of involvement from the patient. The overall goal of patient engagement may not always be about moving to a higher level of engagement and patient empowerment. The study by Hamilton et al outlined a key recommendation of patients valuing the freedom to become gradually more engaged at their own pace. Therefore, desired objectives and the impact of engagement need to be discussed and agreed upon between the organization and the engaged stakeholders. Many health care organizations recognize the varying degrees of engagement. A total of 9 (n=9) citations found in this review referred and/or adapted the participation spectrum outlined by the International Association for Public Participation that identifies different levels of stakeholder and community participation [22]. These findings are relevant not only in the broader health care or health research settings but can be applied to health IT contexts.

### Comparison With Past Work

This scoping review adds to existing literature reviews that have articulated successful engagement characteristics and approaches within the health research context [74]. The impact of engagement can be profound. The review by Manafo et al documented outcomes when engaging patients in health research that include the following: (1) patients feeling empowered, (2) improved trust between researcher and patients, and (3) decreased attrition of study participants. Increased engagement has also positively impacted health IT projects in different ways such as increased usage of health IT, satisfaction with health IT use [35,37,38], and the obtainment of quality/safety-related outcomes [17]. A review on patient involvement in health research by Shippee et al concluded that available literature on patient involvement focused on 1 research phase and particularly earlier stages of research. Similarly, academic studies have involved patients in specific stages of health IT projects, such as the usability testing or the implementation of an inpatient portal and not necessarily throughout all stages of the health IT life cycle. An article by Petersen articulated that despite patients being actively involved in conducting research, patients are often not involved with setting the research agenda, evaluating results, or discussing next steps [20]. Similarly, there is limited evidence reporting or evaluating engagement strategies across multiple phases of a health IT project. As a result, there is a need for further research on sustained engagement throughout different stages of health IT projects and how strategies may differ depending on whether the organization is in a development or implementation phase of a health IT project. This is where identifying findings from the nonhealth IT literature may add value.

Studies have highlighted the important role of health providers in the engagement and activation of patients [57,65]. A study by Graffigna concluded that health providers have a crucial role in influencing the engagement and activation of type 2 diabetic patients in using health IT tools to manage their health condition [75]. Furthermore, several academic and gray literature sources have highlighted the need to incorporate patients and families in underserved populations in health IT initiatives. Involvement and feedback that reflects the diversity of the community can allow organizations to gain a better understanding of the diversity in patient needs [59,68,76,77]. Studies suggested that there were economic and ethnic disparities associated with the use and adoption of health IT [78,79]. Future studies could explore methods in which current engagement strategies can be adapted to effectively engage patients and families from underserved populations. There is also opportunity for health care organizations that primarily work with underserved populations to document and disseminate their strategies in effectively engaging these populations.

Gray literature sources have outlined the importance of evaluating engagement activities to quantify the value of engagement and provide constructive feedback on how engagement initiatives can be improved [63,67]. Evaluation methods that have been recommended include having feedback discussions with patients, providing evaluation forms, and surveys. Other sources have also considered standardizing evaluation by building evaluation components into project plans and leveraging existing standardized tools to evaluate the process and impact of engagement [56,59]. A study by Abelson et al developed a patient engagement evaluation tool that can be used by health care organizations broadly. The evaluation tool consists of 3 unique questionnaires used to evaluate the following 4 evaluation principles: (1) integrity of design and process, (2) influence and impact, (3) participatory culture, (4) collaboration, and (5) common purpose [80].

Despite the number of tools that have been developed to evaluate public and patient engagement, there is a lack of consistency on how engagement strategies are evaluated, and few studies have quantitatively evaluated measures of engagement. A literature review by Esmail et al outlined that there were only 2 studies that evaluated patient engagement within the health research context using quantitative measures [81]. A review by Dukhanin et al identified other existing evaluation tools but concluded that the methodology of each tool varies significantly [82]. Therefore, implications for research include the validation of existing evaluation tools and the combined use of qualitative and quantitative tools to assess engagement. Furthermore, many existing evaluation tools rely heavily on process metrics and measure perceived benefits of engagement [82]. There is a need for health care organizations to increase adoption and use of outcome metrics, such as changes in patient knowledge and attitudes toward engagement, to evaluate patient, public, and community engagement. Meaningful evaluation efforts require capacity and commitment from health care organizations [80]. An implication for health care organizations is to internally build the capacity and culture that supports evaluation efforts to improve the engagement process for patients, families, and organizations.

### Limitations

This review has a few limitations that should be considered when reviewing its findings. The exploration of engagement strategies broadly has led to several considerations and recommendations for how to engage patients and families in health IT initiatives. As identified in the literature, there is a spectrum that exists within the context of patient engagement. Patients can be actively or passively involved in health IT initiatives, and this distinction should be further explored throughout the stages of engagement within the health IT context. A more focused approach that applies to specific health care settings may be completed to solicit results that are appropriate for specific contexts.

Regarding the review methodology, there were challenges with the search strategy regarding the use of broad terms such as theory, model, and frameworks. The terms theory, model, and frameworks were taken from common terminology and approaches used in implementation science [83]. This was done to capture as many different types of engagement tools that could guide future engagement strategies; but as a result, there were a significant number of studies that did not meet the appropriate inclusion criteria for this review but were identified in the initial search. The review included studies published in English only. There is a possibility that relevant studies and resources from health care organizations in non-English speaking countries that have studied or developed engagement strategies in health IT settings were excluded.

### Conclusions

Several studies and gray literature documents identified in this review have reported their findings on successful strategies to engage patients and family members in health care and the positive impact that can emerge when patients and family members are engaged. Several studies have employed a variety of engagement strategies to engage diverse patient populations in health IT projects. Currently, no framework, set of recommendations, or resource document has consolidated all of the lessons learned and considerations to guide health care organizations when engaging patients and family members in a health IT–specific project. There is much to learn and incorporate from the nonhealth IT–specific work that has already been done. With the increasing number of studies reporting their findings of engaging patients in health IT–related initiatives, continuing efforts to evaluate and validate these engagement strategies is needed.

## Appendix

Multimedia Appendix 1 Academic literature data extraction table.

## Abbreviations

EHR: electronic health record
IT: information technology
RQ: research question

## References

[1] (2013). Better Information for Improved Health: A Vision for Health System Use of Data in Canada.

[2] Chang F, Gupta N (2015). Progress in electronic medical record adoption in Canada. Can Fam Physician.

[3] Powell KR, Myers CR (2018). Electronic patient portals: patient and provider perceptions. Online J Nurs Informatics.

[4] Schoen C, Osborn R, Squires D, Doty M, Rasmussen P, Pierson R, Applebaum S (2012). A survey of primary care doctors in ten countries shows progress in use of health information technology, less in other areas. Health Aff (Millwood).

[5] (2014). Institute for e-Health Policy.

[6] Gray BH, Bowden T, Johansen I, Koch S (2011). Electronic health records: an international perspective on 'meaningful use'. Issue Brief (Commonw Fund).

[7] Sulkers H, Tajirian T, Paterson J, Mucuceanu D, MacArthur T, Strauss J, Kalia K, Strudwick G, Jankowicz D (2019). Improving inpatient mental health medication safety through the process of obtaining HIMSS Stage 7: a case report. JAMIA Open.

[8] Kipping S, Stuckey MI, Hernandez A, Nguyen T, Riahi S (2016). A web-based patient portal for mental health care: benefits evaluation. J Med Internet Res.

[9] FitzHenry F, Wells N, Doran J, Hughart K, Levy M, Doulis J (2013). Applying bar code medication administration to make a difference in adverse drug events with potential for harm: lessons learned. Comput Inform Nurs.

[10] (2018). OpenNotes – Patients and Clinicians on the Same Page.

[11] Patel V, Johnson C (2018). HealthIT.

[12] (2017). HealthIT.

[13] Irizarry T, DeVito DA, Curran CR (2015). Patient portals and patient engagement: a state of the science review. J Med Internet Res.

[14] Irizarry T, Shoemake J, Nilsen ML, Czaja S, Beach S, DeVito Dabbs DA (2017). Patient portals as a tool for health care engagement: a mixed-method study of older adults with varying levels of health literacy and prior patient portal use. J Med Internet Res.

[15] (2010). Government of Canada Publications.

[16] Paoletti RD, Suess TM, Lesko MG, Feroli AA, Kennel JA, Mahler JM, Sauders T (2007). Using bar-code technology and medication observation methodology for safer medication administration. Am J Health Syst Pharm.

[17] Strudwick G, Clark C, McBride B, Sakal M, Kalia K (2017). Thank you for asking: exploring patient perceptions of barcode medication administration identification practices in inpatient mental health settings. Int J Med Inform.

[18] Eyasu T, Leung K, Strudwick G (2019). Guiding improvements in user experience: results of a mental health patient portal user interface assessment. Stud Health Technol Inform.

[19] Perfetto EM, Harris J, Mullins CD, dos Reis S (2018). Emerging good practices for transforming value assessment: patients' voices, patients' values. Value Health.

[20] Petersen C (2018). Patient informaticians: turning patient voice into patient action. JAMIA Open.

[21] (2019). Patient-Centered Outcomes Research Institute (PCORI).

[22] (2019). IAP2 Canada.

[23] Arksey H, O'Malley L (2005). Scoping studies: towards a methodological framework. Int J Soc Res Methodol.

[24] Levac D, Colquhoun H, O'Brien KK (2010). Scoping studies: advancing the methodology. Implement Sci.

[25] Häyrinen K, Saranto K, Nykänen P (2008). Definition, structure, content, use and impacts of electronic health records: a review of the research literature. Int J Med Inform.

[26] (2019). Canada Health Infoway: Digital Health in Canada.

[27] Free C, Phillips G, Galli L, Watson L, Felix L, Edwards P, Patel V, Haines A (2013). The effectiveness of mobile-health technology-based health behaviour change or disease management interventions for health care consumers: a systematic review. PLoS Med.

[28] Veritas Health Innovation (2016). Covidence - Better Systematic Review Management.

[29] (2014). Healthcare Information and Management Systems Society.

[30] Walker DM, Hefner JL, Sieck CJ, Huerta TR, McAlearney AS (2018). Framework for evaluating and implementing inpatient portals: a multi-stakeholder perspective. J Med Syst.

[31] Athilingam P, Clochesy JM, Labrador MA (2018). Intervention mapping approach in the design of an interactive mobile health application to improve self-care in heart failure. Comput Inform Nurs.

[32] Lafata JE, Miller CA, Shires DA, Dyer K, Ratliff SM, Schreiber M (2018). Patients' adoption of and feature access within electronic patient portals. Am J Manag Care.

[33] Ackerman SL, Sarkar U, Tieu L, Handley MA, Schillinger D, Hahn K, Hoskote M, Gourley G, Lyles C (2017). Meaningful use in the safety net: a rapid ethnography of patient portal implementation at five community health centers in California. J Am Med Inform Assoc.

[34] Raval MV, Taylor N, Piper K, Thakore M, Hoff K, Owens S, Durham MM (2017). Pediatric patient and caregiver preferences in the development of a mobile health application for management of surgical colorectal conditions. J Med Syst.

[35] Krist AH, Peele E, Woolf SH, Rothemich SF, Loomis JF, Longo DR, Kuzel AJ (2011). Designing a patient-centered personal health record to promote preventive care. BMC Med Inform Decis Mak.

[36] Shapiro-Mathews E, Barton AJ (2013). Using the patient engagement framework to develop an institutional mobile health strategy. Clin Nurse Spec.

[37] Anshari M, Almunawar M, Low PK, Wint Z, Younis MZ (2013). Adopting customers' empowerment and social networks to encourage participations in e-health services. J Health Care Finance.

[38] Greysen SR, Harrison JD, Rareshide C, Magan Y, Seghal N, Rosenthal J, Jacolbia R, Auerbach AD (2018). A randomized controlled trial to improve engagement of hospitalized patients with their patient portals. J Am Med Inform Assoc.

[39] van den Bulck SA, Hermens R, Slegers K, Vandenberghe B, Goderis G, Vankrunkelsven P (2018). Designing a patient portal for patient-centered care: cross-sectional survey. J Med Internet Res.

[40] Wildenbos GA, Maasri K, Jaspers M, Peute L (2018). Older adults using a patient portal: registration and experiences, one year after implementation. Digit Health.

[41] Wildenbos GA, Horenberg F, Jaspers M, Peute L, Sent D (2018). How do patients value and prioritize patient portal functionalities and usage factors? A conjoint analysis study with chronically ill patients. BMC Med Inform Decis Mak.

[42] Metting E, Schrage AJ, Kocks JW, Sanderman R, van der Molen T (2018). Assessing the needs and perspectives of patients with asthma and chronic obstructive pulmonary disease on patient web portals: focus group study. JMIR Form Res.

[43] Carman KL, Dardess P, Maurer M, Sofaer S, Adams K, Bechtel C, Sweeney J (2013). Patient and family engagement: a framework for understanding the elements and developing interventions and policies. Health Aff (Millwood).

[44] (2016). Health Quality Ontario.

[45] Arkind J, Likumahuwa-Ackman S, Warren N, Dickerson K, Robbins L, Norman K, DeVoe JE (2015). Lessons learned from developing a patient engagement panel: an OCHIN report. J Am Board Fam Med.

[46] Boyer AP, Fair AM, Joosten YA, Dolor RJ, Williams NA, Sherden L, Stallings S, Smoot DT, Wilkins CH (2018). A multilevel approach to stakeholder engagement in the formulation of a clinical data research network. Med Care.

[47] Hawke LD, Relihan J, Miller J, McCann E, Rong J, Darnay K, Docherty S, Chaim G, Henderson JL (2018). Engaging youth in research planning, design and execution: practical recommendations for researchers. Health Expect.

[48] Hamilton CB, Hoens AM, Backman CL, McKinnon AM, McQuitty S, English K, Li LC (2018). An empirically based conceptual framework for fostering meaningful patient engagement in research. Health Expect.

[49] Shelef DQ, Rand C, Streisand R, Horn IB, Yadav K, Stewart L, Fousheé N, Waters D, Teach SJ (2016). Using stakeholder engagement to develop a patient-centered pediatric asthma intervention. J Allergy Clin Immunol.

[50] Warren NT, Gaudino JA, Likumahuwa-Ackman S, Dickerson K, Robbins L, Norman K, Lind J, D'Amato S, Foley P, Gold R, Bauer V, Fields SA, Cohen DJ, Clark KD, DeVoe JE (2018). Building meaningful patient engagement in research: case study from ADVANCE clinical data research network. Med Care.

[51] Chung AE, Vu MB, Myers K, Burris J, Kappelman MD (2018). Crohn's and Colitis foundation of America partners patient-powered research network: patient perspectives on facilitators and barriers to building an impactful patient-powered research network. Med Care.

[52] Faulkner M, Alikhaani J, Brown L, Cruz H, Davidson D, Gregoire K, Berdan L, Rorick T, Jones WS, Pletcher MJ (2018). Exploring meaningful patient engagement in ADAPTABLE (aspirin dosing: a patient-centric trial assessing benefits and long-term effectiveness). Med Care.

[53] Coathup V, Teare HJ, Minari J, Yoshizawa G, Kaye J, Takahashi MP, Kato K (2016). Using digital technologies to engage with medical research: views of myotonic dystrophy patients in Japan. BMC Med Ethics.

[54] Suarez-Balcazar Y (2016). Empowerment and participatory evaluation of a community health intervention: implications for occupational therapy. OTJR (Thorofare N J).

[55] (2014). Canadian Institutes of Health Research.

[56] (2016). Health Quality Ontario.

[57] (2013). Government of South Australia: SA Health.

[58] Hailey D (2005). Institute of Health Economics.

[59] (2017). Health Consumers Queensland.

[60] Hayes H, Buckland S, Tarpet M (2012). INVOLVE.

[61] (2017). Health Quality Ontario.

[62] (2013). Paediatric Integrated Cancer Services.

[63] (2018). James Lind Alliance.

[64] (2017). Vancouver Coastal Health.

[65] Center for Advancing Health (2014). American Hospital Association.

[66] (2017). Listowel Wingham Hospitals Alliance.

[67] (2015). Agency for Clinical Innovation.

[68] (2019). Canadian Agency for Drugs and Technologies in Health.

[69] Health Quality Ontario (2016). UW Departments Web Server.

[70] (2018). Canadian Patient Safety Institute.

[71] (2016). NHS England.

[72] (2016). Government of Prince Edward Island.

[73] Arsand E, Demiris G (2008). User-centered methods for designing patient-centric self-help tools. Inform Health Soc Care.

[74] Manafo E, Petermann L, Mason-Lai P, Vandall-Walker V (2018). Patient engagement in Canada: a scoping review of the 'how' and 'what' of patient engagement in health research. Health Res Policy Syst.

[75] Graffigna G, Barello S, Wiederhold B, Bosio AC, Riva G (2013). Positive technology as a driver for health engagement. Stud Health Technol Inform.

[76] Frampton SB, Guastello S, Hoy L, Naylor M, Sheridan S, Johnston-Fleece M (2017). Harnessing evidence and experience to change culture: a guiding framework for patient and family engaged care. NAM Perspectives.

[77] (2018). University of Ottawa Heart Institute.

[78] Abel EA, Shimada SL, Wang K, Ramsey C, Skanderson M, Erdos J, Godleski L, Houston TK, Brandt CA (2018). Dual use of a patient portal and clinical video telehealth by veterans with mental health diagnoses: retrospective, cross-sectional analysis. J Med Internet Res.

[79] Roblin DW, Houston 2nd TK, Allison JJ, Joski PJ, Becker ER (2009). Disparities in use of a personal health record in a managed care organization. J Am Med Inform Assoc.

[80] Abelson J, Li K, Wilson G, Shields K, Schneider C, Boesveld S (2016). Supporting quality public and patient engagement in health system organizations: development and usability testing of the Public and Patient Engagement Evaluation Tool. Health Expect.

[81] Esmail L, Moore E, Rein A (2015). Evaluating patient and stakeholder engagement in research: moving from theory to practice. J Comp Eff Res.

[82] Dukhanin V, Topazian R, DeCamp M (2018). Metrics and evaluation tools for patient engagement in healthcare organization- and system-level decision-making: a systematic review. Int J Health Policy Manag.

[83] Nilsen P (2015). Making sense of implementation theories, models and frameworks. Implement Sci.

