# Supplementary material for: Using Patient and Family Engagement Strategies to Improve Outcomes of Health Information Technology Initiatives: Scoping Review
Source: J Med Internet Res. 2019 Oct 8;21(10):e14683. doi: 10.2196/14683 (PMC6806121; doi:10.2196/14683)
Supplement: Multimedia Appendix 1 [file jmir_v21i10e14683_app1.pdf]

Supplementary Table 1: Academic literature data extraction table

**Study Name**	**Author**	**Publication Year**	**Country of Publication**	**Journal**	**Study design**	**Population Engaged**	**Type of Project**	**Lessons learned**
Meaningful use in the safety net: a rapid ethnography of patient portal implementation at five community health centers in California	Ackerman, S., Sarkar, U., Tieu,. Handley, M., Schillinger, D., Hahn, K., Hoskote, M., Gourley, G., Lyles, C.	2017	USA	Journal of the American Medical Informatics Association	Qualitative	Patients, clinicians	Examination of portal implementation and patient engagement strategies at 5 community health centres	(1) Leveraging trusted staff members and clinicians were effective in encouraging portal use and explaining portal functionalities
Designing a Patient Portal for Patient-Centered Care: Cross-Sectional Survey	Alfons Van den Bulck, S., Hermens, R., Slegers, K., Vandenberghe, B., Goderis, G., Vankrunkelsven, P.	2018	Belgium	Journal of Medical Internet Research	Qualitative	Patients	Exploration of patient needs, expectations and attitudes towards a patient portal	(1) Shared decision making is important; (2) Screening for digital health literacy and providing training to patients could improve patient expectations and attitudes
Adopting Customers' Empowerment and Social Networks to Encourage Participations in E-Health Services	Anshari, M., Almunawar M., Low, P., Wint, Z., Younis M.	2013	Indonesia	Journal of Health Care Finance	Qualitative	Patients	Presentation of model architecture that embeds customer empowerment and social networks in e-health services	(1) Participants prefer to have control over their information; (2) The availability of an online health educator is important to improving the health literacy and empowering patients to be active participants

Lessons Learned from Developing a Patient Engagement Panel: An OCHIN Report	Arkind, J., Likumahuwa-Ackman, S., Warren, N., Dickerson, K., Robbins, L., Norman, K., DeVoe, J.	2015	USA	Journal of the American Board of Family Medicine	Qualitative	Patients	Development of patient engagement panel for community health information network	(1) Patients who select themselves to participate in engagement panels are not average patients and come with drive and direction; (2) Brief, simple descriptions of expected role, time commitment and potential benefits for engagement is beneficial; (3) Necessary costs for members include food, travel compensation, reimbursement for time and training opportunities (4) It is important to engage patients early; (5) Patients were interested in opportunities to attend and present at research conferences; (7) A customized compensation model can provide flexibility to what is received as a token of appreciation for patient participation
User-centered methods for	Arsand, E., Demiris, G.	2008	Norwa y	Informatics for Health & Social Care	Other	Patients	Summary of methods to facilitate patient	(1) Scenarios and storytelling are effective ways of explaining how

designing patient-centric self-help tools							involvement in the design process of e-health tools	a technical solution works for patients and caregivers; (2) Time for several meetings is needed to allow users to understand the possibilities that technology provides; (3) Use interviews to gain an in-depth understanding of the patient experience
Intervention Mapping Approach in the Design of an Interactive Mobile Health Application to Improve Self-care in Heart Failure	Athilingam, P., Clochesy, J., Labrador, M.	2017	USA	Computers, Informatics, Nursing	Qualitative	Patients, clinicians	Design of a mobile self-care management intervention for patients with heart failure	(1) Patient engagement is needed for health behaviour change; (2) The use of internet, e-mail and text messaging can be leveraged for communication and long-term engagement with patients
A Multilevel Approach to Stakeholder Engagement in the Formulation of a Clinical Data Research Network	Boyer, A., Fair, A., Joosten, Y., Dolor, R., Williams, N., Sherden, L., Stallings, S., Smoot, D., Wilkins, C.	2018	USA	Medical Care, American Public Health Association	Other	Patients, clinicians, community members	Formulation of a clinical data research network	(1) engage stakeholders early in the planning process; (2) provide stakeholders with clear expectations; (3) provide adequate preparation (orientation, training, resources) for both the stakeholders and the academic team members; (4) prioritize effective

								communication with regular updates and provide explanation of acronyms and research/medical terminology; (5) use established networks of stakeholder groups; (6) actively engage leaders of patient and health advocacy groups; and (7) leverage providers as trusted agents
Designing a Patient-Centered User Interface for Access Decisions about EHR Data: Implications from Patient Interviews	Caine, K., Kohn, S., Lawrence, C., Hanania, R., Meslin, E., Tierney, W.	2014	USA	Journal of General Internal Medicine	Qualitative	Patients	Design of a user interface that records patient decisions about accessing electronic health data	(1) Provide flexibility when using interview methods to discuss ideas and topics that were deemed important by patients; (2) Engaging patients in the design phase of an electronic health record system; (3) Patients revealed the importance of policies and guidelines that need to be put in place for the privacy and control over health information
Patient And Family Engagement: A Framework For Understanding The Elements And Developing	Carman, K., Dardess, P., Maurer, M., Sofaer, S., Adams, K.,	2013	USA	Health Affairs	Qualitative	Patients and family members	Presentation of a patient engagement model that can occur throughout	(1) Individual factors can potentially impact a patients' willingness and ability to engage with the healthcare system such as health literacy,

Interventions And Policies	Bechtel, C., Sweeney, J.						the healthcare system	education; (2) Healthcare organizations and staff can encourage patient engagement through demonstrating that patient participation and leadership is imperative to the achievement of quality improvement goals. (3) Policies and practices create expectations that patients can and will serve as advisers and decision makers on committees and patient-centred councils; (3) Policy makers can create mechanisms so that patients can provide input; (5) Funding mechanisms can also be created requiring patient participation in the decision-making process
Crohn's and Colitis Foundation of America Partners Patient-Powered Research Network	Chung, A., Vu, M., Myers, K., Burris, J., Kappelman, M.	2018	USA	Medical Care, American Public Health Association	Qualitative	Patients	Discovery of patient needs on building patient-centred research networks	(1) Patients wanted to feel like equal partners in every phase; (2) Patients were more likely to sustain long-term participation if they felt invested in the

								outcomes; (3) Patients felt it was information was important when it was communicated by their doctor; (4) Patients desired regular check-ins and vested interest; (5) Patients felt that transparency about how their research contributions are being used and by whom when providing data for research.
Using digital technologies to engage with medical research: views of myotonic dystrophy patients in Japan	Coathup, V., Teare, H., Minari, J., Yoshiawa, G., Kaye, J., Takahasi, M., Kato, K.	2016	Japan	BMC Medical Ethics	Mixed methods	Patients	Exploration of patients' views and attitudes to using an electronic patient registry	(1) Patients were interested in being engaged through digital platforms but wanted to maintain control over the interaction; (2) A majority of patients wanted to be informed about when and how their research data is being shared.
Anything but engaged: user involvement in the context of a national electronic health record implementation	Cresswell, K., Morrison, Z., Crowe, S., Robertson, A.,Sheikh A.	2011	UK	Informatics in Primary Care	Qualitative	Hospital staff, community stakeholders	Evaluation of the implementation of EHR software	(1) Engagement efforts were negatively affected by a lack of user involvement in implementation timelines; (2) Allocate sufficient time for as much organic engagement to occur as

								possible; (3) Draw on effective individuals that span between management and clinical settings
Exploring Meaningful Patient Engagement in ADAPTABLE (Aspirin Dosing: A Patient-centric Trial Assessing Benefits and Long-term Effectiveness)	Faulkner, M., Alikhaani, J., Brown, L., Cruz, H., Jones, W., Pletcher, M.	2018	USA	Medical Care, American Public Health Association	Other	Patients	Implementation of a patient-centric trial of Aspirin dosing	(1) Patients were most productive when supported with dedicated sessions; (2) it is important to keep patients informed of the trial progress, and eliciting feedback; (3) It is best to start engaging patients early and provide opportunities for patients to have input at the formative stages; (4) Patients benefited from being engaged as a large group (more than 2-3 patients) so that they can encourage each other and produce strength from shared discussion and ideas.
Introducing the patient's perspective in hospital health technology assessment (HTA): the views of HTA producers, hospital	Gagnon, M., Desmartis, M., Gagnon, J., St-Pierre, M., Gauvin, F., Rhainds, M., Lepage-Savary,	2012	Canada	Health Expectations	Qualitative	Patients, hospital managers, health technology assessment producers	Exploration of patient involvement in health technology assessments	(1) Patients emphasized the importance of involving patients throughout the assessment process to improve patients' knowledge and give

managers and patients	D., Coulombe, M., Tantchou Dipankui, M., Legare, F.							them a sense of responsibility in the decision making process; (2) It is important to have a clear definition of the objectives of patient involvement; (3) Give patients' feedback; (4) Clearly define patients' role and what is expected of them; (5) Have a minimum of 2 patient representatives on committees; (6) Provide basic training to patients to improve their background knowledge
Electronic screening for lifestyle issues and mental health in youth: a community-based participatory research approach	Goodyear-Smith, F., Corter, A., Suh, H.	2016	New Zealand	BMC Medical Informatics and Decision Making	Qualitative	Patients , clinicians	Evaluation of a community-based participatory research approach to implementing a youth programme for electronic screening and intervention for mental health issues	(1) Consultation with patients is improved when patients are empowered, given time to reflect; (2) Provide various modes of communication to keep youth engaged

A randomized controlled trial to improve engagement of hospitalized patients with their patient portals	Greysen, S., Harrison, J., Rareshide, C., Magan, Y., Seghal, N., Rosenthal, J., Jacolbia, R., Auerbach, A.	2018	USA	Journal of the American Medical Informatics Association	Quantitative	Patients	Trial of a tablet-based educational intervention to increase patient engagement with patient portals	(1) Training is crucial to improve patient engagement activities
An empirically based conceptual framework for fostering meaningful patient engagement in research	Hamilton, C., Hoens, A., Backman C., McKinnon, A., McQuitty, S., English, K., Li, L.	2018	Canada	Health Expectations	Other	Patients	Presentation of a conceptual framework to foster meaning patient engagement	(1) Participants wanted to be informed about the expectations associated with their roles in the project; (2) Participants valued being gradually more engaged at their own pace; (3) Participants preferred the use of jargon free language; (4) Participants wanted to be reimbursed for their time and contributions; (5) Engage patients in groups, ideally more than one so that patients can learn and support each other; (6) Participants outlined value of convenience, flexibility to choose how and when to contribute; (7) Participants valued the opportunity to access meetings

								remotely; (8) Participants valued financial support that covered expenses related to engagement; (9) Training is valued to understand the language and process of project
Engaging youth in research planning, design and execution: Practical recommendations for researchers	Hawke, L., Relihan, J., Miller, J., McCann, E., Rong, J., Darnay, K., Docherty, S., Chaim, G., Henderson, J.	2018	Canada	Health Expectations	Other	Patients	Recommendations on how to integrate youth in research projects led by academic researchers	(1) It is important to engage youth from the specific demographic that the research project is primarily targeting; (2) Formally recognize youth contributions through honoraria or co-authorship on published papers; (3) Clearly communicate roles and responsibilities of youth; (4) Be transparent about expected project timelines, expected number of meetings and amount of commitment; (5) Conduct meetings in an easily accessible and comfortable environment; (5) Identify concepts that may be confusing and explain them in jargon-

								free terms; (6) Having pre-brief and debrief sessions for youth provides the opportunity to check in and provide mentorship; (7)Make it known how patient input has impacted the project or decisions made in the project
Implementation of youth-adult partnership model in youth mental health systems research: Challenges and successes	Heffernan, O., Herzog, T., Schiralli, J., Hawke, L., Chaim, G., Henderson, J.	2017	Canada	Health Expectations	Other	Patients	Development, implementation and success of a youth-adult partnership initiative	(1) Develop partnership early in the planning process of the project; (2) Develop guidelines for youth engagement and recognize potential organizational barriers; (3) There is a need for training for both youth and adult involved in partnership; (4) Establish a method of recruitment that can engage a diverse group of individuals
Youth engagement in the YouthCan IMPACT trial	Henderson, J., Hawke, L., Relihan, J.	2018	Canada	Canadian Medical Association Journal	Other	Patients	Evaluation of a youth engagement model in a randomized controlled trial	1) A strong youth facilitator was important to successfully facilitate advisory group meeting; (2) Clearly define patient goals and vision to ensure that engagement is

								meaningful for all parties involved
Engaging Primary Care Patients to Use a Patient-Centered Personal Health Record	krist, A., Woolf, S., Bello, G., Sabo, R., Longo, D., Kashiri, P., Etz, R., Loomis, J., Rothemichm, S., Peele, J., Cohn, J.	2014	USA	Annals of Family Medicine	Qualitative	Patients, clinicians	Evaluation of the feasibility of primary care practices in engaging patients to use a patient portal	(1) Adoption of a team-approach to engaging patients positively influenced the uptake of the patient portal
Patients' Adoption of and Feature Access Within Electronic Patient Portals	Lafata, J., Miller, C., Shires, D., Dyer, K., Ratliff, S., Schreiber, M.	2018	USA	The American Journal of Managed Care	Qualitative	Patients	Description of patients' adoption and feature access of an online patient portal	(1) It is important to provide alternative channels for vulnerable patient populations to be informed and engaged
Assessing the Needs and Perspectives of Patients With Asthma and Chronic Obstructive Pulmonary Disease on Patient Web Portals: Focus Group Study	Metting, E., Jantine Schrage, A., Kocks, J., Sanderman, R., van der Molen, T.	2018	The Netherl ands	Journal of Medical Internet Research Formative Research	Qualitative	Patients	Exploration of patient needs and opinions to facilitate the development of patient web portals	(1) Portal use can be improved by providing information on the portal in plain language. (2) Training of patients through web-based tutorials can be used; (3) Training activities are recommended for healthcare professionals to effectively communicate with patients

Emerging Good Practices for Transforming Value Assessment: Patients' Voices, Patients' Values	Perfetto, E., Harris, J., Mullins, C., dosReis, S.	2018	USA	Value in Health	Qualitative	Patients, caregivers	Collection of first-hand experiences with patient engagement in value assessments	(1) Engage early on in the process and continuously, in the development stage and not just in a review capacity; (2) Engage a range of patients and patient groups to understand a variety of needs; (3) Utilize patient-provided information and existing outreach mechanisms to collect information from patients; (4) Be transparent with patients, document and publicize all suggestions received during an open-comment period; (5) Appreciate and accommodate patient community resource constraints; (6) Clearly define how and how often patients can make contact to engage (objectives, expectations, roles, number of hours, compensation and anticipated deliverables); (7) Provide incentives to

								collaborate with patients; (8) Acknowledge and disclose real and potential conflicts of interest by all patient participants.
eHealth Literacy: Patient Engagement in Identifying Strategies to Encourage Use of Patient Portals Among Older Adults	Price-Haywood, E., Harden-Barrios J., Ulep, R., Luo, Q.	2017	USA	Population Health Management	Qualitative	Patients	Examination of relationship between portal usage and literacy to encourage technology adoption	(1) Participants expressed concerns about information security, lack of personalization, lack of resources and desire for skills and technical support; (2) Communicating value of engagement is important; (3) Addressing computer literacy can alleviate concerns about patient self-efficacy; (4) It is important to provide patient training
Pediatric Patient and Caregiver Preferences in the Development of a Mobile Health Application for Management of Surgical Colorectal Conditions	Raval, M., Taylor, N., Piper, K., Thakore, M., Hoff, K., Owens, S., Durham, M.	2017	USA	Journal of Medical Systems	Qualitative	Patients, healthcare professionals	Engagement of patients and caregivers in the development of a colorectal disease management application	(1) The engagement of the clinician team was crucial to the success of engaging patients; (2) Clinical team was available during the planning stages of the research project; (3) It would be beneficial if

								clinicians can be trained on patient engagement methods
Developing medical device technologies from users' perspectives: A theoretical framework for involving users in the development process	Shah, S., Robinson I., AlShawi, S.	2009	UK	International Journal of Technology Assessment in Health Care	Other	Healthcare professionals, patients, caregivers	Proposal of a theoretical framework for involving different end users in the development process of medical device technologies	(1) Common methods to engage users include interviews, focus groups, brainstorming sessions and users-producers seminars at the concept stage; (2) Interviews, usability tests and users' feedback is suggested at the design stage; (3) Usability tests, interviews and discussion at testing and trial stages; (4) Ethnography, interviews and surveys are suggested for deployment and post-deployment stages of the device development process
Using the Patient Engagement Framework to Develop an Institutional Mobile Health Strategy	Shapiro-Mathews, E., Barton, A.	2013	USA	Clinical Nurse Specialist	Other	Patients	Development of an institutional strategy for mobile health technologies	(1) An interdisciplinary, team-based approach is required; (2) A clinical nurse specialist was important to providing patient education and informing the design of mobile applications that

								meet the needs of patients
Using stakeholder engagement to develop a patient-centered pediatric asthma intervention	Shelef, D., Rand, C., Streisand, R., Horn, I., Yadav, K., Stewart, L., Foushee, N., Waters, D., Teach, S.	2016	USA	Clinical Nurse Specialist	Qualitative	Patients, caregivers	Results of stakeholder engagement in the development of research protocols	(1) It is crucial to build trust with stakeholders; (2) It is important to provide stakeholders with resources and support needed to contribute in a meaningful way; (3) Smaller group meetings with caregivers alone were conducted to develop trusting relationships and stakeholders to provide input in a focused way; (4) Stakeholders contributed most significantly when presented with very specific questions and draft materials; (5) Engaging a trusted community partner can be helpful in understanding what information, resources and approaches would help community stakeholders feel valued; (6) financial resources are needed to

								support the time and effort commitment by stakeholders
Empowerment and Participatory Evaluation of a Community Health Intervention: Implications for Occupational Therapy	Suarez-Balcarzar, Yolanda	2005	USA	Occupational Therapy Journal of Research	Other	Community members	Description of an empowerment and participatory model applied to a health technology project	(1) Community members received training in the project; (2) Clinical team can play an important role in increasing the capacity for community members as leaders
Integrating community-based participatory research and informatics approaches to improve the engagement and health of underserved populations	Unertl, K., Schaefbauer, C., Campbell, T., Senteio, C., Siek, K., Bakken, S., Veinot, T.	2015	USA	Journal of the American Medical Informatics Association	Other	Community members	Evaluation of a community-based participatory research approach to an informatics project	(1) Strategies to incorporate perspectives included focusing on the families rather than individuals; (2) Focus on trust as a factor incorporated into the engagement of community members when designing research projects.
Framework for Evaluating and Implementing Inpatient Portals: a Multi-stakeholder Perspective	Walker, D., Hefner, J., Sieck, C., Huerta, T., McAlearney, A.	2018	USA	Journal of Medical Systems	Qualitative	Patients, clinicians, hospital staff	Presentation of a framework for evaluating patient portal implementations	(1) Patients revealed patient specific outcomes that should be factored when evaluating the success of a portal implementation; (2) Training and technology

								support for patients are critical
Building Meaningful Patient Engagement in Research	Warren, N., Gaudino, J., Likumahuwa-Ackman, S., Dickerson, K., Robbins, L., Norman, K., Lind, J., D'Amato, S., Foley, P., Gold, R., Bauer, V., Fields, S., Cohen, D., Clark K., DeVoe, J.	2018	USA	Medical Care, American Public Health Association	Other	Patients	Development of an engagement framework for a clinical data research network	(1) Develop policies around stakeholder compensation; (2) providing advisory roles and responsibility bring a level of respect to positions
How do patients value and prioritize patient portal functionalities and usage factors? A conjoint analysis study with chronically ill patients	Wildenbos, G., Horenberg, F, Jaspers, M., Peute, L., Sent, D.	2018	The Netherl ands	BMC Medical Informatics and Decision Making	Qualitative	Patients	Analysis of patients' preferred usage factors and patients' prioritization of portal functionalities	(1) Patients prefer information in simple language with limited jargon.
Older adults using a patient portal: registration and experiences, one year after implementation	Wildenbos, G., Maasri, K., Jaspers, M., Peute, L.	2018	The Netherl ands	Digital Health	Qualitative	Patients	Exploration of older adult patient experiences using a patient portal	(1) Health literacy level of patients was also a strong factor that influenced the interest and ability to use the patient portal; (2) There should be a focus to train and support

								healthcare providers in their interaction with patients

Supplementary Table 2: Grey literature data extraction table

Name of Resource	Source	Date of publication	Country of Publication	Relevant Field	Recommendations/Considerations
Patient Experience and Consumer Engagement: A Framework for Action	Agency for Clinical Innovation, Australia	2015	Australia	Healthcare	(1) Develop relationships with consumer and community based organizations<br>(2) Orient consumers and what it means to be engaged<br>(3) Provide feedback and evaluation of patient experience and engagement<br>(4) Encourage the use of performance measures to demonstrate value of engagement initiatives<br>(5) Match consumer needs to organizational needs<br>(6) Organize specific events, education and training to support consumers to develop skills

					(7) Provide opportunities for consumers to get involved in conferences, events and other committees<br>(8) Provide reimbursement for expenses incurred by consumers during engagement activities
Consumer Involvement in Health Technology Assessment	Alberta Heritage Foundation for Medical Research	2005	Canada	Health Technology Assessments	(1) Roles of consumers are documented and agreed between researchers and consumers involved<br>(2) Budget appropriately for consumers to be reimbursed for their involvement<br>(3) Access to training and personal support for consumers involved<br>(4) Ensure researchers have necessary skills to involve consumers in research process<br>(5) Consumers are involved in decisions about how participants are recruited and informed about the research process<br>(6) Acknowledge consumer involvement and provide details to describe how consumers were involved in research reports<br>(7) Involve consumers in choosing the methods used to distribute and disseminate research findings<br>(8) Interact with consumer and patient organizations to provide knowledge and useful contacts
Patient, Family, Caregiver and Public Engagement Framework	British Columbia Ministry of Health	2018	Canada	Healthcare	(1) Develop trust-based relationships to achieve individual, community and system goals<br>(2) Use co-design techniques to involve all stakeholders in the design process<br>(3) commit to respect, dignity and listening to understand with stakeholders<br>(4) Engagement requires resources such as time, money and training<br>(5) Clarify objectives of engagement

					(6) Clarify in advance how patients', families' and caregivers' views will influence the decision making process<br>(7) Report the results of engagement activities helping others learn
CADTH Framework for Patient Engagement in Health Technology Assessment	CADTH	2019	Canada	Health Technology Assessments	(1) Honoraria and travel expenses are provided to patient and stakeholder committee members<br>(2) Training is provided on assessment methodologies and implementation of patient engagement strategies in the health technology assessment process<br>(3) Use multiple methods of communication such as email, social media platforms (Twitter and Facebook) to solicit patient input and feedback<br>(4) Track and update clear timelines of each milestone<br>(5) Share patient perspectives on the CADTH website<br>(8) Provide thank you letters, along with feedback and suggestions for future involvement to each patient group that contributes to the assessment process
Strategy for Patient-Oriented Research (SPOR) Patient Engagement Framework	Canadian Institutes of Health Research	2014	Canada	Research	(1) Involve patients in key governance structures and decision-making processes<br>(2) Clarifying roles and expectations<br>(3) Integrate patient engagement into training strategies, including with research/trainees/interns and opportunities for patients<br>(4) Support patients to share in the responsibility of mentoring other patients<br>(5) Implement tangible incentives to encourage participation of patients

Engaging Patients in Patient Safety	Canadian Patient Safety Institute	2018	Canada	Healthcare	(1) Allow patients partners to boards, steering committees, quality and safety committees, patient and family advisory councils and patient groups<br>(2) Create reporting structures that solicit patient feedback, compliments, complaints and reported incidents<br>(3) Create a patient role that responsible for recruiting and training additional patient partners<br>(4) Budgets for financial resources are needed as compensation for patient partners
A New Definition of Patient Engagement: What is Engagement and Why is it Important?	Centre for Advancing Health	2010	USA	Healthcare	(1) Establish a trusting relationship between healthcare professionals and patients<br>(2) It is important to allow for opportunities to improve patient knowledge<br>(3) Provide a communication forum where questions and issues can be brought up and explanations can be provided
Guide for Engaging with Consumers and the Community	Government of South Australia	2013	Australia	Healthcare	(1) Communication should be open, respectful and culturally appropriate<br>(2) Feedback should be heard, acknowledged and used<br>(3) Training should be provided to clinicians and staff to ensure engagement is consumer focused<br>(4) Processes that define consumer and community role in planning stages is important<br>(5) Identify objectives and impact on relevant stakeholders<br>(6) Identify communication plan, resources and methods<br>(7) Evaluate outcomes against specified objectives

Consumer and Community Engagement Framework	Health Consumers Queensland	2017	Australia	Healthcare	(1) Engagement should take place across planning, implementation, monitoring and evaluation of projects<br>(2) Consumers should be involved in processes from as early as possible<br>(3) Consumers are engaged at the planning phase of projects<br>(4) Consumers and staff are provided with clear information about the engagement activities, the scope and responsibilities of their roles<br>(5) All staff and consumers are provided with training on consumer and community engagement, how to partner effectively and communicate<br>(6) Engagement partners have opportunities for professional development<br>(7) Evaluation of engagement activities is built into project plans<br>(8) Reimbursement and remuneration processes are in place to ensure consumers are remunerated for their time<br>(9) Creative engagement methods are explored to ensure activities are appropriate for all parts of the community
Engagement Toolkit	Health PEI	2016	Canada	Healthcare	(1) Inform patients using websites, pamphlets and fact sheets<br>(2) Consult patients with focus groups, surveys and public meetings<br>(3) Involve patients through advisory groups, working groups and workshops<br>(4) Collaborate with patients using advisory committees, retreats and planning workshops<br>(5) Empower patients with think tanks and study groups

Patient Partnering Framework	Health Quality Ontario	2017	Canada	Healthcare	(1) Clinical staff can be leveraged to inform patients and empower patients to make decisions<br>(2) A community advisory group can be responsible for engaging community members, staff and healthcare professionals to understand stakeholder needs at the organization.<br>(3) Patients and family members can be engaged in hiring committees through their involvement in screening applicants, conducting interviews and selecting appropriate candidates
Choosing Methods for Patient and Caregiver Engagement: A Guide for Health Care Organizations	Health Quality Ontario	n.d.	Canada	Healthcare	(1) One on one interviews can be used to explore an issue in depth with a single patient or caregiver, hear from patients and caregivers who may be unlikely to participate in groups<br>(2) Group discussions can be used to gather multiple perspectives on an issue and discuss a range of issues that affect a broad audience<br>(3) Surveys can be used to gather systemic feedback on focused questions from a large number of people<br>(4) Anonymous comment boxes can be used to solicit feedback on sensitive issues<br>(5) Patient and Caregiver Advisory Councils can be used when high-priority, long-term decisions need to be made within an organization and when significant relationship building between an organization and its clients are needed<br>(6) Committees, task forces can be used when various perspectives need to be drawn for a single project<br>(7) Meetings with patient/caregiver groups and associations can be used to recruit patient and caregivers to be involved in organizational projects<br>(8) Storytelling can be used to build empathy around an issue and draw from lived experiences to address a topic or issue<br>(9) Experience based co-design can be used to partner with patients and caregivers in designing services and programs at the organization

Engaging with Patients and Caregivers about Quality Improvement	Health Quality Ontario	n.d.	Canada	Healthcare	(1) Engage an experienced patient champion who can help engage other patients<br>(2) Engage two or more patients<br>(3) Clearly define patient roles contributions and expectations in advance to address any concerns of inexperience or nervousness<br>(4) Plan to protect patient privacy<br>(5) Clearly articulate the type of patient information, who will see the information and why that information is being shared<br>(6) Allow multiple channels of communication for feedback and discussion<br>(7) Engage local community or social service organizations to recruit patients and family members from specific populations<br>(8) Provide engaged patients with background readings to prepare prior to meetings<br>(9) Enable patients to be involved by covering for child care, elder care, parking, travel expenses and accommodation.<br>(10) Frequently check in with patients for any questions or additional information need<br>(11) Evaluate engagement activities by leveraging standardized tools to assess planning, execution and impact of engagement in the six months post-engagement<br>(12) Provide patients with feedback on impact of their input and any constraints that may have hindered the implementation of their recommendations

Briefing notes for researchers: public involvement in NHS, public health and social care research	INVOLVE Coordinating Centre	2012	UK	Research	(1) Involve people at an early stage so that they feel part of the research and also have a sense of ownership of the research<br>(2) Define and clarify why stakeholders can getting involved, their roles, time commitment and what they can expect from the engagement team<br>(3) Write in jargon-free language and accommodate for any accessibility needs<br>(4) Budget for expenses incurred by stakeholders while being involved in project (time, travel, childcare, training, translation, attendance to conferences/events)<br>(5) Offer training and support for stakeholders through informal or formal mechanisms<br>(6) Acknowledge the contribution that the public have made when writing journal articles and reports<br>(7) Explore ways to meet people in their own environments and ensure that meeting places are accessible to all who are interested in attending
Quality & Patient Engagement Framework	Listowel Wingham Hospitals Alliance (CA)	n.d.	Canada	Healthcare	(1) Organizational surveys can be used to measure experience and respond to feedback<br>(2) Patient and family meetings can be used to share information<br>(3) Patient feedback should be solicited in development of educational materials<br>(4) A robust family presence policy is important to meaningfully engage patients<br>(5) Collaborating with patients as partners on the care teams<br>(6) Sharing patient stories at every opportunity

Toolkit for e-Mental Health Implementation	Mental Health Commission of Canada	2018	Canada	Healthcare	(1) Ensure engagement methods are culturally sensitive and appropriate<br>(2) Outline training and support provided to patients during engagement<br>(3) Create and clarify a communication plan for engaging patients
The James Lind Alliance Guidebook	National Institute for Health Research Evaluation, Trials and Studies Coordinating Centre University of Southampton	2018	UK	Research	(1) When engaging external organizations, it is encouraged that the organization is contacted by a known contact<br>(2) An initial kick off meeting can be used to explain the idea of the engagement, answer questions<br>(3) Early discussions with stakeholders regarding their capacity to participate and preferences for ongoing communication is key to ensuring that stakeholders are informed throughout the process<br>(4) A communication plan can be used throughout the engagement process to communicate data collection methods, and research results<br>(5) A survey can be administered in advance to identify any potential safeguarding issues for working with patients who are vulnerable or at risk<br>(6) Allocation of financial resources to reimburse participants for travel expenses and their time<br>(7) Provision of information or a telephone call in advance of a meeting may be helpful<br>(8) Provide evaluation forms for patients and care partners to provide constructive feedback on their experience anonymously<br>(9) Engage patients and care partners to talk through the format of meetings beforehand

Patient Engagement in Patient Safety: A Framework for the NHS	NHS	2016	United Kingdom	Patient Safety	(1) Healthcare professionals play a significant role in encouraging patients to engage<br>(2) Take into consideration the amount of effort/time/resources required for patients to engage<br>(3) Having a consistent team involved in patient engagement can foster trusting relationships and a common purpose
A Toolkit for Consumer Participation and Engagement	Paediatric Integrated Cancer Service (AUS)	2013	Australia	Healthcare	(1) It is important to include a range of cultural and linguistically diverse backgrounds<br>(2) Evaluation is an important step to ensuring engagement activities are meaningful<br>(3) Consider potential barriers for patients during engagement activities<br>(4) Set up a database with consumer contact information and areas of interest<br>(5) Cover expenses incurred by consumers for engaging in activities<br>(6) Provide opportunity to use teleconference to attend meetings<br>(7) Provide opportunity to attend training and educational sessions<br>(8) Run an annual consumer forum specific to a topic<br>(9) Provide a regular newsletter to update consumers
Engagement Rubric for Applicants	Patient-Centred Outcomes Research Institute	2016	USA	Research	(1) Involve stakeholders in developing the research questions and desired outcomes to ensure that the project is useful and important to the stakeholders<br>(2) Having stakeholders participating in the recruitment of study participants, drafting study materials/protocols<br>(3) Stakeholders participating in the evaluation of patient and stakeholder engagement<br>(4) Stakeholders can serve as a representative on a data safety monitoring board

					(5) Stakeholders can identify partner organizations for dissemination of study results<br>(6) Stakeholders can participate in dissemination efforts through authoring manuscripts, presenting study findings to difference audiences
Patient Engagement Framework	University of Ottawa Heart Institute	n.d.	Canada	Healthcare	(1) Solicit patient feedback through surveys, group discussions<br>(2) Provide patients and families with a formalized role working alongside clinical or research staff if desired<br>(3) Engage patients and families through committees, patient and family-centre programs allowing them to advise on operational and policy development<br>(4) Support patients through training and support
Community Engagement Framework	Vancouver Coastal Health	n.d.	Canada	Healthcare	(1) Determine the appropriate level of engagement that meets your goals<br>(2) Tailor the format and method of engaging stakeholders to suit the context<br>(3) Identify and communicate with all key stakeholders including staff and management, community partners, possible steering or standing<br>advisory committees, etc. when developing an engagement process for a project<br>(4) Develop a communication plan for sharing findings about engagement process<br>(5) Clarify roles and contributions of community participants<br>(6) Commit to sending a report to participants about how their input was utilized

					(7) Evaluate if re-engagement of individuals in making recommendations or implementing changes is valuable
